# Development of Intermittent Fever by Obstructed Drains

**Published:** 1874-05

**Authors:** G. J. Fisher


### Development of Intermittent Fever at Sing Sing, N. Y., by Obstructed Drains.

In the year 1866 or 1867, a culvert on the Hudson River Railroad was obstructed by dumping rock and sand, which detained a great deal of vegetable and animal matter within an estuary or pond of an acre or more in extent. This resulted in the development of intermittent fever among a number of families living adjacent to this exceedingly impure and stagnant water. Being at the time Assistant Sanitary Inspector for the town of Ossining, I reported this nuisance to the Metropolitan Board of Health; and when the "order" for its abatement was obeyed by the Railroad Company, by reopening the culvert to the free entrance and exit of the tides, it resulted in stamping out the fever and ague. The second exception was first observed three or four years ago, when a considerable number of houses—say over thirty—were erected in the southern end of Spring and State streets. Parallel with, and lying between those streets, is a valley, which has never been well drained; but of late the drainage has been so defective as to amount to obstruction; to which must be added the slops, surface drainage, and subsoil, of all these houses, yards, and privies, which has resulted in the development of sufficient malaria to have caused a number of cases of intermittent fever in the immediate neighborhood, where the disease was previously unknown.

Proper drainage would doubtless promptly restore the original salubrity of this locality.

It appears to me that these examples furnish the most positive proof of the direct production of malaria from defective drainage —or, rather, no drainage.

Injury to the health and destruction of the lives of our citizens, where the causes are obvious and preventable, renders a careless and culpably ignorant board of town, village or city officials responsible and, at least in equity, if not in law, actionable for damages as much as a private corporation for like damages to health and life by defective construction of bridges or railway tracks, steam boilers or rickety tenement houses.—*G. J. Fisher, Sanitarian for June.*

